# Serine proteases and metalloproteases are highly increased in irritable bowel syndrome Tunisian patients

**DOI:** 10.1038/s41598-023-44454-3

**Published:** 2023-10-16

**Authors:** Souha Soussou, Amin Jablaoui, Vincent Mariaule, Aicha Kriaa, Houda Boudaya, Magdalena Wysocka, Ali Amouri, Ali Gargouri, Adam Lesner, Emmanuelle Maguin, Moez Rhimi

**Affiliations:** 1 Microbiota Interaction With Human and Animal Team (MIHA), Micalis Institute-UMR1319, AgroParisTech, Université Paris-Saclay, INRAE, 78350 Jouy-en-Josas, France; 2 Laboratory of Molecular Biology of Eukaryotes, Center of Biotechnology of Sfax, University of Sfax, Sfax, Tunisia; 3 Department of Gastroenterology, Hedi Chaker University Hospital, Sfax, Tunisia; 4 Faculty of Chemistry, University of Gdansk, Gdańsk, Poland

**Keywords:** Irritable bowel syndrome, Proteases

## Abstract

Serine proteases are involved in many biological processes and are associated with irritable bowel syndrome (IBS) pathology. An increase in serine protease activity has been widely reported in IBS patients. While most of the studies focused on host proteases, the contribution of microbial proteases are poorly studied. In the present study, we report the analysis of proteolytic activities in fecal samples from the first Tunisian cohort of IBS-M patients and healthy individuals. We demonstrated, for the first time, that metalloproteases activities were fourfold higher in fecal samples of IBS patients compared to controls. Of interest, the functional characterization of serine protease activities revealed a 50-fold increase in trypsin-like activities and a threefold in both elastase- and cathepsin G-like activities. Remarkably, we also showed a fourfold increase in proteinase 3-like activity in the case of IBS. This study also provides insight into the alteration of gut microbiota and its potential role in proteolytic modulation in IBS. Our results stressed the impact of the disequilibrium of serine proteases, metalloproteases and gut microbiota in IBS and the need of the further characterization of these targets to set out new therapeutic approaches.

## Introduction

Irritable bowel syndrome (IBS) is considered one of the most common functional gastrointestinal disorders that affect bowel habits and alters patients' mental health and wellbeing^1,2^. IBS can be classified based on the predominant bowel habit into constipation IBS (IBS-C), diarrhea (IBS-D) and mixed or alternating IBS (IBS-M or IBS-A) where the patient has mixed bowel habits^3^. IBS global prevalence is around 11%, varying from 5 to 10% in most European countries, China, and the United States^1,4^. In Africa, the prevalence of IBS is also high; however, there is a noteworthy lack of data as available African studies are primarily hospital-based or concern specific populations, stressing the need for proper cohort-based studies^5,6^. The annual healthcare costs of IBS could be raised to billions of dollars, constituting a burden on the healthcare system^7^. With unsettled exclusive biomarkers^8^, IBS is hardly diagnosed and clinicians rely mainly on symptom based-diagnosis, according to the revised Rome IV criteria^3,9,10^. Symptomatic treatments for IBS are the only available remedies as no efficient curative therapy has been reported so far^11^. IBS is a multifactorial syndrome whose etiology is far from being understood. However, the scientific community gained more insight into this syndrome and shed light on two major imbalances affecting the gut microbiota and the global proteolytic activity^12,13^.

Proteases account for approximately 2% of the human genome and are involved in many important biological functions, from digestion to nervous signaling and immunity defenses^14^. A diverse repertoire of proteases of host and gut microbiota origins is secreted in the intestinal tract. Tight regulation of such proteolytic activities is mandatory to prevent tissue injury. Shifted balance of proteases or their specific inhibitors can induce disruption of the intestinal barrier function and neuronal sensitization reported in IBS^15^. A significant increase in the total protease activity in fecal and colonic supernatants from IBS patients has been reported^16–20^. Among the enhanced proteolytic activities, cysteine- and serine-like activities were reported to be the most upregulated^13,19,20^. Serine proteases are responsible for the cleavage of epithelial tight-junctions such as ZO-1 and Occludin, and are known to target protease activated receptors (PAR) omnipresent on epithelial cells and neurons^18,21^. PAR-1, 2, and 4 are the main receptors involved in the epithelial signaling in digestive diseases; meanwhile, PAR1 is associated to the nerve signaling pathways in IBS^19,20^. The overexpression of proteases was thereby inferred to be causing inflammation by increasing intestinal permeability, signaling and activating nociceptive and enteric neurons, which induce hyperalgesia^22^. Most studies on IBS have been focused on host proteases as reflected by the recent identification of the Trypsin-3 isoform as major source of trypsin activity in IBS colonic tissue^19^. However, the identity of upregulated serine protease and their respective contribution to the overall fecal proteolytic activity still need to be unveiled.

In the present study, we constituted the first Tunisian cohort of IBS patients and analyzed their fecal proteolytic activities distribution. Our results demonstrate that total proteolytic activity is increased in IBS patients compared to healthy controls. Study of fecal protease activities shows that serine proteases are the most increased proteases and that trypsin-like and, for the first time, protease 3-like activities were upregulated in IBS patients. Remarkably, we demonstrate that metalloproteases are highly overactive in IBS patients. We also highlight that dysbiotic gut microbiota composition is significantly correlated with upregulated proteolytic activities in IBS patients.

## Methods

### Ethics declaration

This study was performed in line with the principles of the Declaration of Helsinki. All human experiments were performed with the approval of ethic committee under the ethical approval number: CPP SUD No. 0203/2019. Informed consent was obtained from all participants before recruitment to the study.

### IBS-M cohort construction

This cohort was generated in the Department of Gastroenterology at the CHU Hedi CHAKER in Sfax-Tunisia. After obtaining their consent, volunteers were subjected to clinical exams, including radiography and endoscopy and IBS was diagnosed conforming to the Rome IV criteria^3,10^. Participants’ medical records were verified to confirm their eligibility according to inclusion and exclusion criteria^23^. These exclusion criteria included (1) the use of probiotics, prebiotics or antibiotics in the last 6 months, (2) diagnosis or treatment of digestive inflammation or bacterial/parasitic infection and (3) other severe diseases or history of alcohol or drug abuse. The study’s final cohort comprises 51 IBS-M patients against 31 healthy controls recruited randomly; their demographic data are shown in Table 1. Fecal samples were collected, transferred and stored at − 80 °C under same conditions.

**Table 1:** Patients’ demographic data.

Group	Number	Sex	Age mean (SEM)
Healthy	31	20F/11M	40.2 (3.7)
IBS-M	51	31F/20M	43.5 (2.9)

### Fecal water samples preparation

To prepare fecal waters, 200 mg of thawed feces were added to 1 ml of assay buffer (20 mM Tris–HCl, 200 mM NaCl, pH 7.8). The samples underwent a 1 min sonication (10 s on/10 s off, amplitude 39%) and were centrifuged at 4 °C, 10 000 rpm for 20 min. Supernatants were then harvested and filtered using a 0.8 µM Nalgene® sterile filter. Fecal water samples were prepared freshly for protease activity assays.

### Proteolytic activity assays

The proteolytic activity assessment consists of using specific chromogenic or fluorogenic substrates to monitor each of the specified activities detailed in Table 2^24–27^. Total activity was quantified using azocasein as substrate and the reaction was stopped with 10% (w/v) added trichloroacetic acid (TCA, Sigma). All reactions were performed in Tris–HCl buffer (20 mM Tris–HCl, 200 mM NaCl, 1 mM CaCl2, pH 7.8). Fecal supernatants were diluted to the appropriate concentration and 20 µl of the dilution were added to the reaction mixture containing 20 µl of the suitable substrate (Table 2) in a final volume of 200 µl. The reactions were then incubated on 96-well plates for 30 min at 37 °C. Absorbance (410 nm) or fluorescence (Excitation: 360 nm, Emission: 460 nm) were measured using a Perkinelmer® plate reader at room temperature. We considered that 1 unit of protease activity refers to the quantity of enzyme catalyzing the degradation of 1 µM of substrate or the release of 1 µM of product per minute in these conditions.

**Table 2:** Specific substrates (0.3 mg/ml) and inhibitors (Cysteine-, HNE-, CatG-like: 10 µM; Metalloproteases and Serine-like: 1 mM; Trypsin-like: 100µM; Chymotrypsin- and PR3-like: 0.1 µM).

Type	Specificity	Sequence
Substrates	Cysteine-like	AB2-Ile-Leu-Pro-Glu-ANB-NH2 (Chromogenic)
	Metalloprotease-like	MCA-Lys-Pro-Leu-Gly-Leu-DNP-Dpa-Ala-Arg-NH2 (Fluorogenic)
	Trypsin-like	AB2-Val-Val-Ser-Lys-ANB-NH2 (Chromogenic)
	Chymotrypsin-like	AB2-Lys-His-Trp-Tyr-ANB-NH2 (Chromogenic)
	HNE-like	AB2-Met-Pro-Val-Ala-Trp-Glu-Tyr-(3-NO2)-NH2 (Fluorogenic)
	PR3-like	AB2-Tyr-Tyr-ABU-Asn-Glu-Pro-Tyr-(3-NO2)-NH2 (Fluorogenic)
	CatG-like	MCA-Phe-Val-Thr-Gnf-Ser-Trp-AB2-NH2 (Fluorogenic)
	MMP-2-like	MCA-Pro-Leu-Ala-Nva-Dap(DNP)-Ala-Arg-NH2 (Fluorogenic)
	MMP-9-like	Dabcyl-APFEMSAK(FAM)-NH2 (Fluorogenic)
Inhibitors	Cysteine-like	E-64
	Metalloproteases	EDTA
	Serine-like	PMSF
	Trypsin-like	Soybean Trypsin Inhibitor (STI)
	Chymotrypsin-like	Nα-Tosyl-Phe Chloromethyl Ketone (TPCK)
	HNE-like	N-(Methoxysuccinyl)-Ala-Ala-Pro-Val-chloromethyl ketone
	PR3-like	Bt-Val-Tyr-Asp-nValP(O-C6H4-4-Cl)2
	CatG-like	Ac-Phe-Val-Thr-PhgP(4-guanidine)-(OC6H4-4-S-Me)2

### Inhibition assays

To estimate each protease family among the total proteolytic activity, inhibition assays were achieved using selective inhibitors of serine proteases, cysteine proteases and metalloproteases that are phenylmethanesulfonyl fluoride (PMSF, 1 mM), E-64 (10 µM) and EDTA (1 mM), respectively (Table 2). Other inhibitors were also used to determine trypsin-like (100 µM), Chymotrypsin-like (Cmt, 0.1 µM), Human Neutrophil Elastase-like (HNE, 10 µM), Proteinase 3-like (PR3, 0.1 µM), and Cathepsin G-like (CatG, 10 µM) activities (Table 2). Protein concentrations were measured with Nanodrop (LabTech) at 280 nm (1 absorbance unit = 1 mg/ml).

### DNA extraction and microbiota profiling

To analyze fecal microbiota composition and richness, total DNA was extracted from 200 mg of feces from each subject conforming to Godon et al.^28^ described protocol. DNA amplification was performed during 30 amplification cycles at 65˚C with primer pairs for the V3-V4 fragments of the 16S ribosomal RNA locus (F343 5′CTTTCCCTACACGACGCTCTTCCGATCTACGGRAGGCAGCAG′3 and R784 5′GGAGTTCAGACGTGTGCTCTTCCGATCTTACCAGGGTATCTAATCCT′3). The obtained amplicons (510 bp) were then purified and undertook a second 12 cycles PCR by adding a 6 bp index to the reverse primer R784 (AATGATACGGCGACCACCGAGATCTACACTCTTTCCCTACACG AC) and by using a modified reverse primer (CAAGCAGAAGACGGCATACGAGAT-indexGTGACTGGAGTTCAGACGTGT) via the Illumina Miseq technology. The purification and loading of the amplification products onto the Illumina MiSeq cartridge were fulfilled according to the manufacturer’s instructions. To analyze raw sequences, we used the bioinformatics pipeline FROGS (Find Rapidly OTU with Galaxy Solution)^29^. Sequences were clustered into 97% similarity operational taxonomic units (OTUs) with a cut-off value of 0.03, then classified using a naive Bayesian method against the Silva Ribosomal Database with an 80% confidence interval. OTU sequence data were computed to relative abundance ± SEM. α and β diversity analysis was accomplished with the phyloseq R package. Observed, Shannon and Simpson indices were used to evaluate α-diversity. Community composition profiles between groups were evaluated using permutational multivariate analysis of variances (PERMANOVA). Kruskal–Wallis test and Dunn’s test were applied to analyze the differences in phyla, families, and genera^30^. Benjamini–Hochberg corrections (BH) were used to avoid false positives (significance threshold = 0.05)^31^. The pairwise correlations between each specific genus and host parameters were calculated using the Spearman's nonparametric rank correlation coefficient. The R 3.3.1 software, along with the corrplot and Hmisc packages, were used to generate the Spearman correlation matrix.

### Statistical analyses

Data are expressed as means ± SEM. GraphPad Prism 9.0 (GraphPad software, Inc.) was used for statistical analysis. Differences in proteolytic activities between IBS patients and controls were determined using the Kruskal–Wallis test followed by the Dunn test for multiple comparisons. For inhibition assays, we compared the proteolytic activity in the presence or absence of inhibitors using the Mann–Whitney test. Statistical significance is indicated as *p < 0.05, **p < 0.01, and ***p < 0.001. Statistical significance was considered at *p* value < 0.05.

## Results and discussion

IBS is a high-prevalence disorder that constitutes a worldwide concern, with annual medical costs averaging billions of dollars in industrialized and Western populations^32^. In African and Asian developing countries, the IBS prevalence is also estimated to be considerably high with dramatically increasing incidence, nevertheless very limited epidemiologic reports are available^33–35^. Data regarding IBS in Africa and Tunisia, in particular, are scarce^36,37^. The gap in statistical data in these countries could reflect poor healthcare systems, limited access to medical cares, or deficient/not updated medical records databases^1^. Previous studies explored proteolytic profiles and human gut microbiota composition in IBS cohorts mainly from the Western population. The study of African IBS cohorts would constitute an important step in tracking any differences due to geographic, genetic or cultural diversities^36–38^. In the present work, we built the first Tunisian IBS cohort. Collected fecal samples from 51 IBS-M patients and 31 healthy volunteers were subjected to a proteolytic characterization using specifically engineered substrates and inhibitors as well as fecal microbiota profiling.

### Proteases profiling in IBS fecal samples

**Figure 1:**
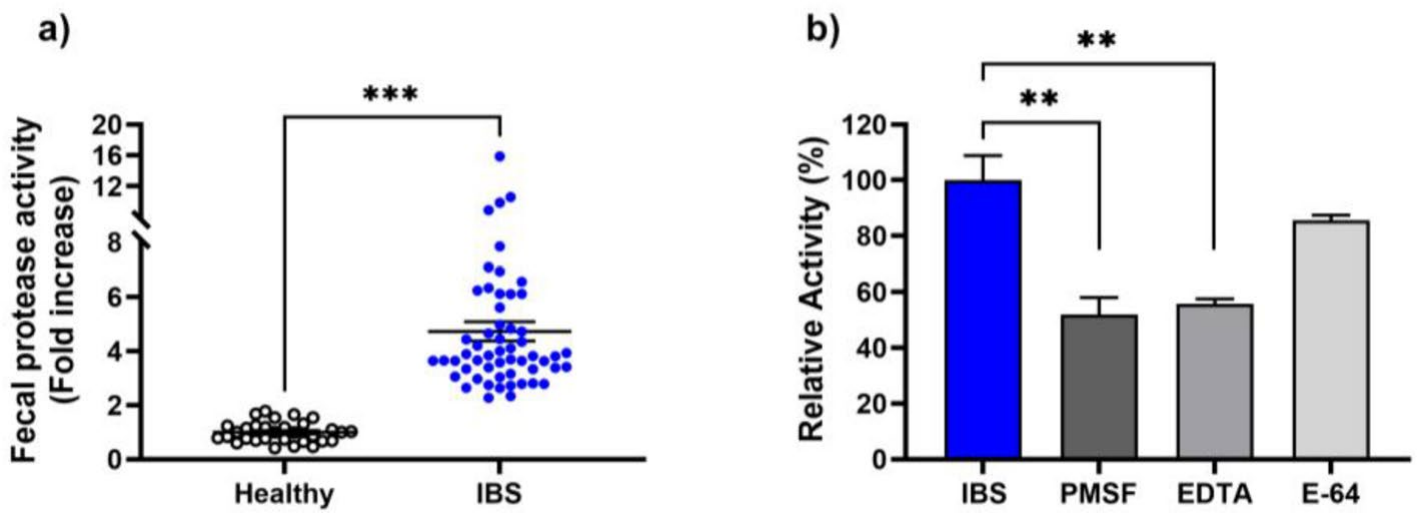
Measurement of the total proteolytic activity in fecal waters of healthy controls (N = 31) and IBS patients (N = 51). (**a**) Total protease activity in fecal samples of healthy individuals and IBS patients. (**b**) The relative protease activity with or without inhibition with PMSF, EDTA and E-64 in IBS samples. Data are mean ± SEM. The total protease activity was analyzed by Mann Whitney test in healthy and IBS patients. The relative protease activity corresponds to the percentage of the measured activity in a given sample reported to the mean activity measured in IBS patients’ samples which was considered 100% (IBS = 24.5 ± 0.26 U/mg). Kruskal–Wallis test followed by Dunn’s test were performed to compare the relative activity in presence and absence of inhibitors. **p < 0.01, ***p < 0.001.

A dysregulation of proteolytic activities has been shown to contribute to several digestive inflammatory disorders including IBS. In this study, we characterized the profile of proteolytic activities in Tunisian IBS patients. For this purpose, we first assessed protease activity in fecal waters and showed that total protease activity was about fourfold higher in IBS patients than in healthy controls (Fig. 1a, Table 2). To determine the respective contribution of protease families to the increased proteolytic activities, several specific protease inhibitors were tested (Fig. 1b, Table 2). Interestingly, the proteolytic activity was significantly reduced by 49.1% in IBS samples (*p* < 0.01) in the presence of PMSF, a broad-spectrum serine protease inhibitor, demonstrating a major contribution of serine proteases. In the presence of the metalloprotease inhibitor EDTA, proteolytic activity was also significantly reduced by 44.2% (*p* < 0.01). When using cysteine protease inhibitor E-64, we observed only 16% decrease of the activity, although not statistically significant (Fig. 1b). Such data demonstrate that serine proteases and metalloproteases were the major contributors to the increased protease activities in IBS patients. These findings are consistent with prior studies reporting a significant increase in serine protease activity in IBS patients compared to healthy controls^17,19,21,39^. In contrast, previous studies did not investigate the upregulation of metalloproteases in IBS. Interestingly, several studies described the involvement of metalloproteases in other digestive inflammation including inflammatory bowel diseases^40^.

**Figure 2:**
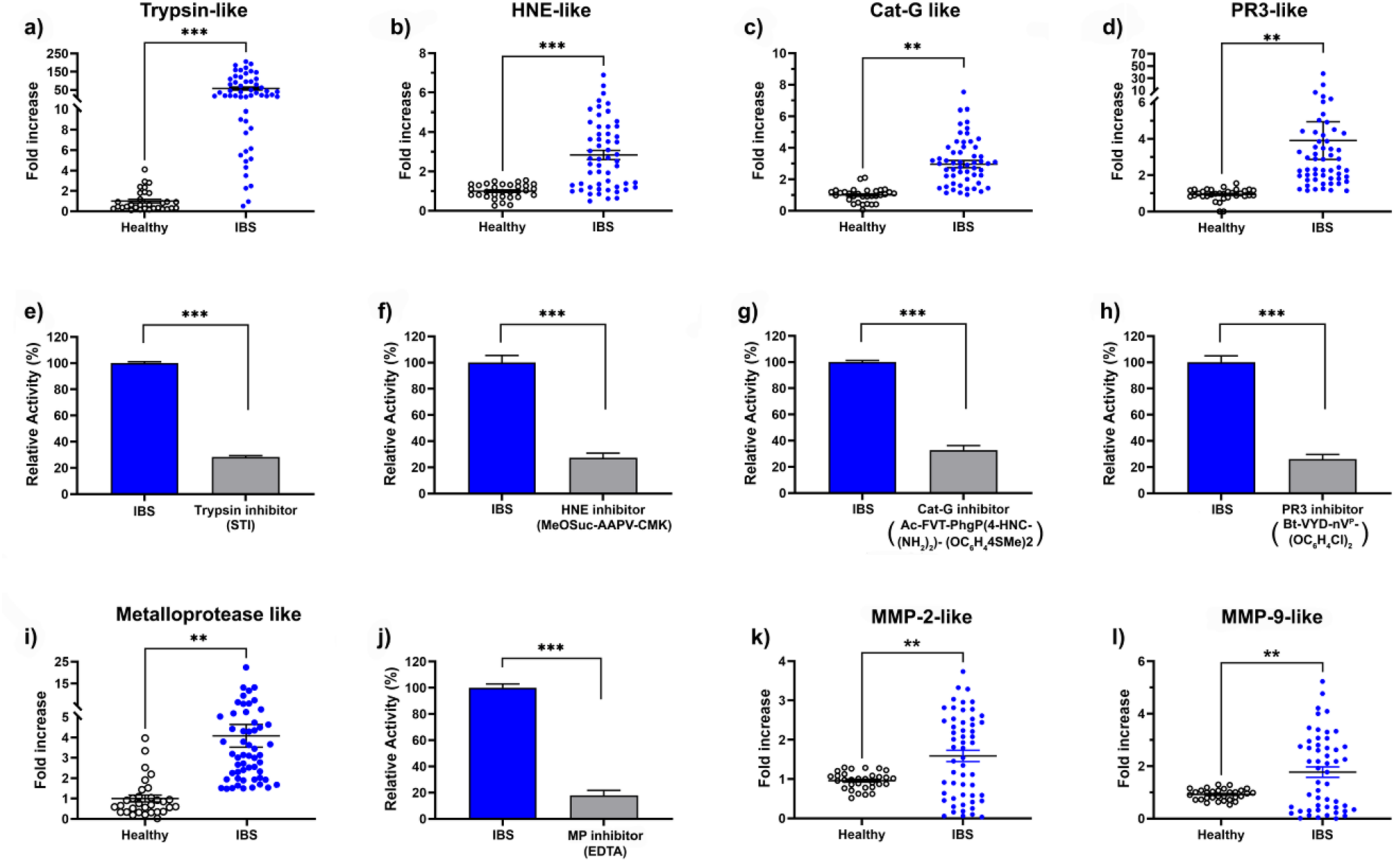
Characterisation of protease activities in fecal samples using specific substrates and inhibitors. (**a**) Fecal trypsin-like activity in healthy controls (N = 31) and IBS patients (N = 51). (**b**) HNE-like activity in fecal samples of healthy individuals and IBS patients. (**c**) Fecal Cat-G-like activity in healthy controls and IBS patients. (**d**) PR3-like activity in healthy subjects and IBS patients. (**e**) Trypsin-like activity in absence and in presence of its specific inhibitor. (**f**) Inhibition assays of HNE like-activity with the HNE specific inhibitor. (**g**) Inhibition assays of CatG-like activity using specific inhibitor. (**h**) PR3-like activity without and in the presence of its specific inhibitor. (**i**) Fecal metalloprotease activity in healthy and IBS patients. (**j**) Fecal metalloprotease activity in presence and absence of its inhibitor. (**k** and **l**) Fecal MMP-2-like and MMP-9-like activities in healthy and IBS patients, respectively. The relative activity represents the measured activity divided by the maximal activity defined as 100%. Error bars represent SEM. Data were analyzed using the Mann Whitney test. **p < 0.01, ***p < 0.001.

To further analyze the overactive serine proteases in IBS fecal samples, we used several substrates and inhibitors specific to serine protease subfamilies known to be associated with gut inflammation^41–43^. As shown in Fig. 2a, trypsin-like activity was 50-fold higher in IBS patients than healthy subjects (*p* < 0.001). We also assessed HNE- and CatG-like activities in IBS fecal samples and both showed a threefold increase compared with healthy subjects (*p* < 0.001 and *p* < 0.01 respectively) as shown in Fig. 2b,c. Regarding the PR3-like activity, we showed a nearly fourfold increase in IBS samples compared to controls (Fig. 2d). These results were confirmed by the use of specific inhibitors of each targeted serine protease subfamily. In fact, using specific inhibitors, fecal trypsin-, HNE-, CatG-, and PR3-like activities were significantly decreased to 27.3 (*p* < 0.001; Fig. 2e), 27.2 (*p* < 0.001; Fig. 2f), 32.7 (*p* < 0.01; Fig. 2g) and 26% respectively (*p* < 0.001; Fig. 2h) in IBS samples. As for serine proteases, we sought to assess metalloprotease activity. As shown in Fig. 2i, the measured metalloprotease activity showed a fourfold increase in the fecal supernatants from IBS patients compared to those of healthy subjects (*p* < 0.01). In the presence of EDTA, such activity was significantly reduced to 18% (*p* < 0.001; Fig. 2j). Further analysis demonstrates that fecal MMP-2 and MMP-9 were 1.5 and 1.8 fold-higher in IBS-M subjects, respectively (Fig. 2k, l). It is worth noting that, observed decrease of metalloprotease activity may reflect the contribution of both metalloproteinase and trypsin-like proteases as EDTA inhibitor will also impact calcium ions that stabilize trypsin active conformation. This scenario remains unlikely given the high selectivity of the used substrate towards metalloproteases^44^. We further, explored the specificity of the used substrates. For example we measured the fecal HNE activity using its specific substrate and in presence of the HNE inhibitor we observed a significant decrease of the HNE activity (Fig. 2f). However, when we used PR3 inhibitor or CatG inhibitor or EDTA no significant decrease of the activity was observed stressing the specificity of the HNE substrate in our experiments. The same experience was conducted in case of each SP (PR3, CatG) and metalloproteases, and same results were obtained confirming the specificity of used substrates under operating conditions (data not shown)*.* Of note, chymotrypsin activity was also measured but no statistically significant differences were detected between the samples of IBS and healthy subjects (data not shown).

Based on our results, trypsin-like activity is the most increased in IBS-M patients compared to healthy individuals. A previous report showed a 55-fold increase of fecal trypsin-like activity in American IBS-C patients^21^. Furthermore, an increase in trypsin-like activity (about twofold) was shown in IBS-M patients’ colonic biopsies^19^. Such marked difference (about 50 vs. twofold) between biopsies, reflecting host proteases and fecal samples encompassing both host and microbial proteases, raises the question of the contribution of microbial proteases in the context of IBS. The increase of the HNE-like activity (threefold higher) observed in the samples of the Tunisian cohort studied herein was also described in the case of American IBS-C patients with a stronger effect (about 32-fold increase)^21^. In contrast, Gecse et al. did not find significant differences in HNE-like activity between Hungarian IBS-M also called IBS-A and healthy subjects^17^. For CatG-like activity, our data showed a two-fold increase in patients compared to healthy control. In contrast, a previous study on fecal CatG activity concluded at the lack of significant difference between IBS-D versus healthy subjects^45^.

To the best of our knowledge, this is the first study showing an upregulation of the PR3-like and metalloprotease activities in IBS. We may hypothesize that in IBS patients PR3 activities can be associated to visceral sensitivity through the activation of MMP already described^46,47^. Further mechanistic studies are needed to investigate the role of PR3 in IBS physiopathology. Moreover, we previously demonstrated that fecal PR3-like activity is also increased in IBD patients compared to healthy subjects stressing the key role of PR3 in digestive inflammation^48^. For metalloprotease, many studies highlighted the role played by such enzymes in nociception and hyperalgesia. In fact, human MMP-2 and MMP-9 are involved in the mediation of pain hypersensitivity^49,50^. Interestingly, increased MMP activities have been reported in colonic biopsies from patients suffering from IBD, a gastrointestinal disease characterized by abdominal pain^51,52^. However, our previous study did not find a significant difference in fecal metalloprotease activities between IBD patients and healthy subjects^48^. This presumably indicates the crosstalk between MMP produced by the host and their corresponding microbial inhibitors.

### Fecal microbiota analysis

**Figure 3:**
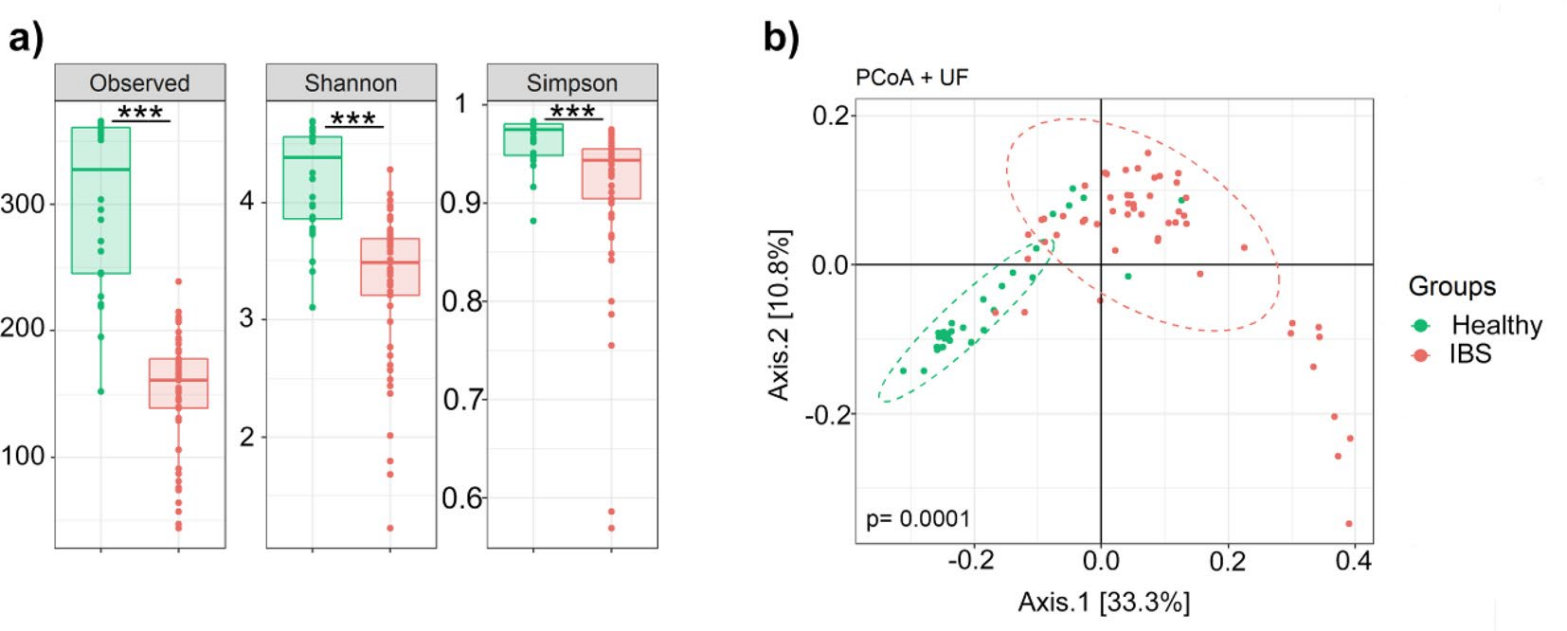
Microbiota composition of IBS patients in comparison to healthy controls. (**a**) Alpha diversity indices. Patients with IBS (red) have lower microbial diversity than healthy controls (green) on observed operational taxonomic units (OTU), Shannon and Simpson tests (*p* < 0.001). (**b**) Beta diversity index. Principal coordinate analysis (PCoA) revealed that IBS patients have altered microbial clustering compared to healthy controls as shown by unweighted UniFrac algorithms of the beta diversity (*p* = 0.0001). Statistical significance was determined using the Mann Whitney and PERMANOVA tests for alpha and beta diversity respectively.

A significant modification of the gut microbiota composition in IBS patients compared to healthy subjects was noted. Microbiota analysis in feces stressed a marked decrease in bacterial abundance and richness in IBS samples compared to those of healthy controls based on Shannon and Simpson tests (*p* < 0.001) (Fig. 3a). Beta diversity analysis, based on the Principal Coordinate Analysis (PCoA), unraveled a distinctly altered clustering of the gut microbiota between IBS and healthy fecal samples (PERMANOVA; *p* = 0.0001) (Fig. 3b). Our results are coherent with previous studies assessing the gut microbiota in both IBS and healthy subjects where the overall abundance of bacteria, but not necessarily diversity, was decreased in IBS patients^12,32,34,35,53^.

**Figure 4:**
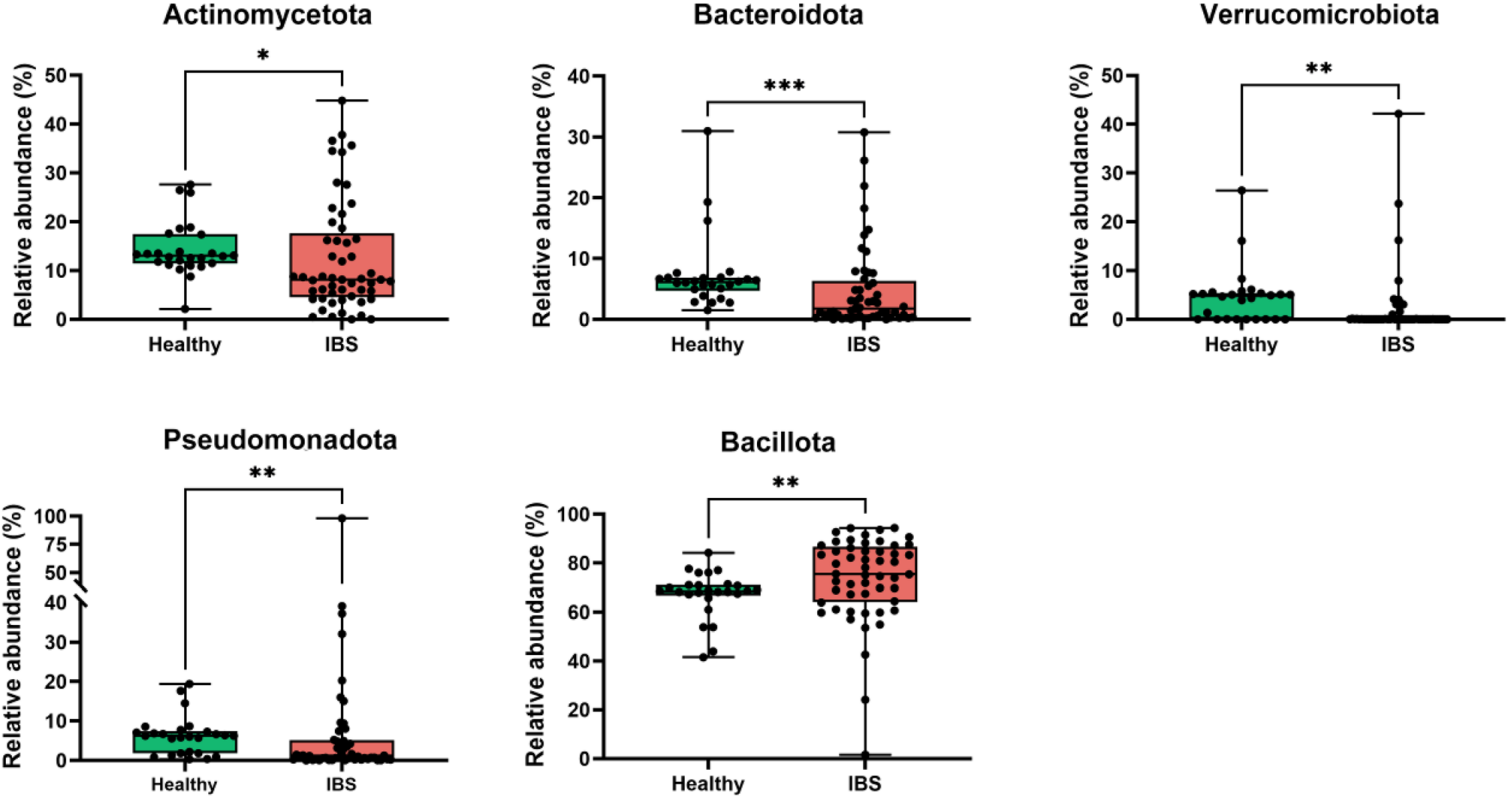
Phyla richness and distribution. Relative abundance (percentage) of bacterial communities at phyla level in IBS samples (red) compared to healthy controls (green). Statistical significance was assessed using the Mann Whitney test. *p < 0.05, **p < 0.01, ***p < 0.001.

As shown in Fig. 4, the IBS patients displayed more *Bacillota* (previously *Firmicutes*) than controls (*p* < 0.01) and a significant decrease of *Bacteroidota* (previously *Bacteroidetes*) (*p* < 0.001) and thus an increased *Bacillota*:*Bacteroidota* ratio. We also observed a significant decrease in *Verrucomicrobiota, Pseudomonatota* (previously *Proteobacteria*) (*p* < 0.01) and *Actinomycetota* (previously *Actinobacteria*) phyla (*p* < 0.05) in IBS patients. These data support previously reported bacterial phyla modulated in IBS patients compared to healthy subjects^54,55^. It is worth noting the discrepancies between *Actinomycetota* and *Pseudomonatota* phyla abundance observed between IBS cohorts^54,56–60^.

**Figure 5:**
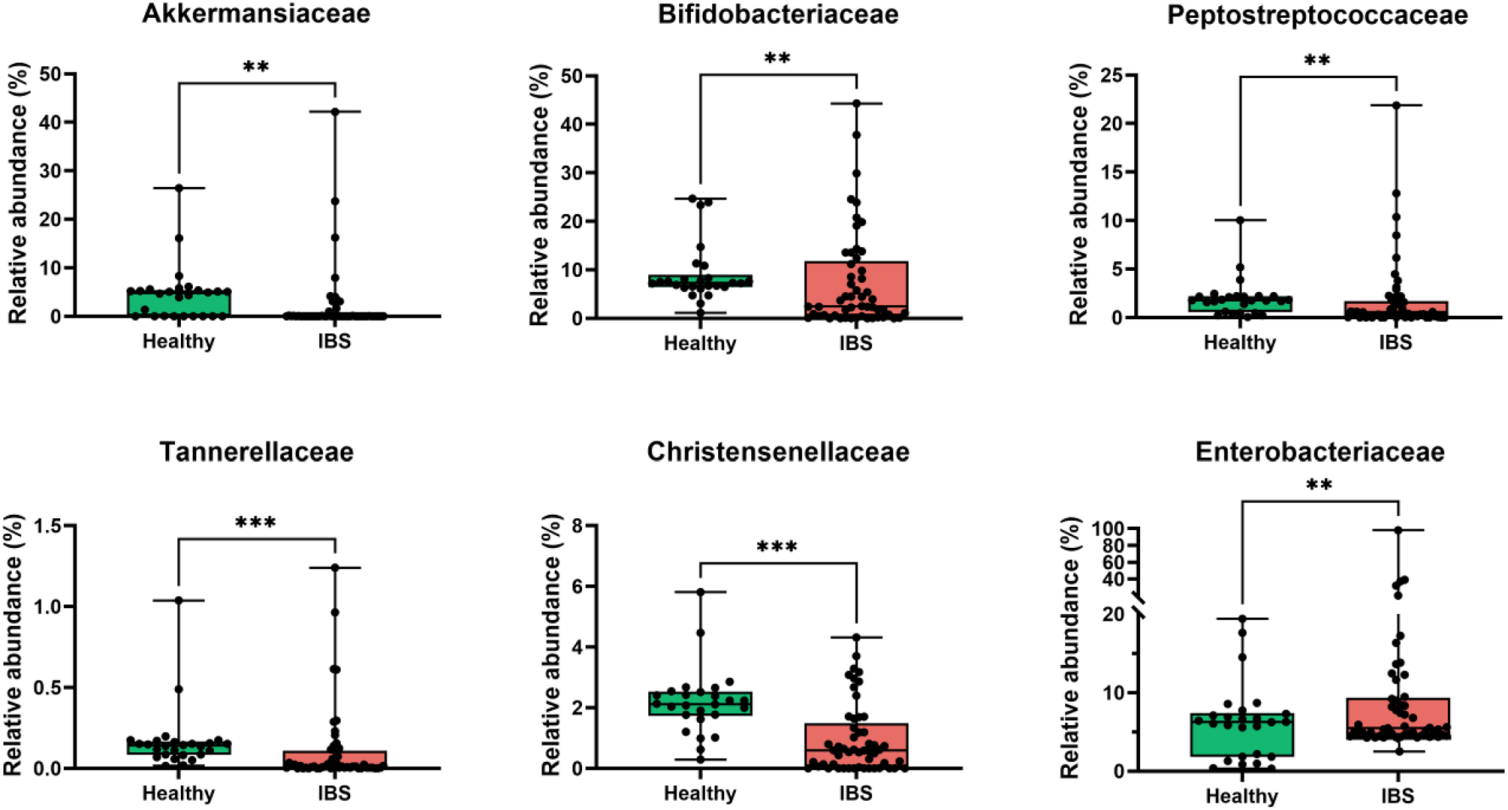
Differentially abundant bacterial families from IBS stools (red) compared to healthy ones (green). Mann Whitney test was applied to analyze statistical differences. **p < 0.01, ***p < 0.001.

At family level, the comparative analysis displayed in IBS patients a significant decrease of five bacterial families *Bifidobacteriaceae* (*p* < 0.01)*, Akkermansiaceae* (*p* < 0.01), *Christensenellaceae* (*p* < 0.001), *Tannerellaceae* (*p* < 0.001) and *Peptostreptococcaceae* (*p* < 0.01) (Fig. 5). From depleted families identified in IBS patients, some are associated with health benefits (e.g., *Bifidobacteriaceae, Christensenellaceae* and *Akkermansiaceae*). In fact, a positive correlation between *Christensenellaceae* depletion and longer transit time or constipation has been described, as well as its association with other gastrointestinal disorders, including IBD^61^. In contrast, the *Enterobacteriaceae* family showed a strong increase of their relative abundance in IBS samples compared to controls. Enrichment of *Enterobacteriaceae* encompassing known pathogenic species with pro-inflammatory potential is commonly observed in patients with IBS, suggesting their potential contribution to the disorder progression^62^.

**Figure 6:**
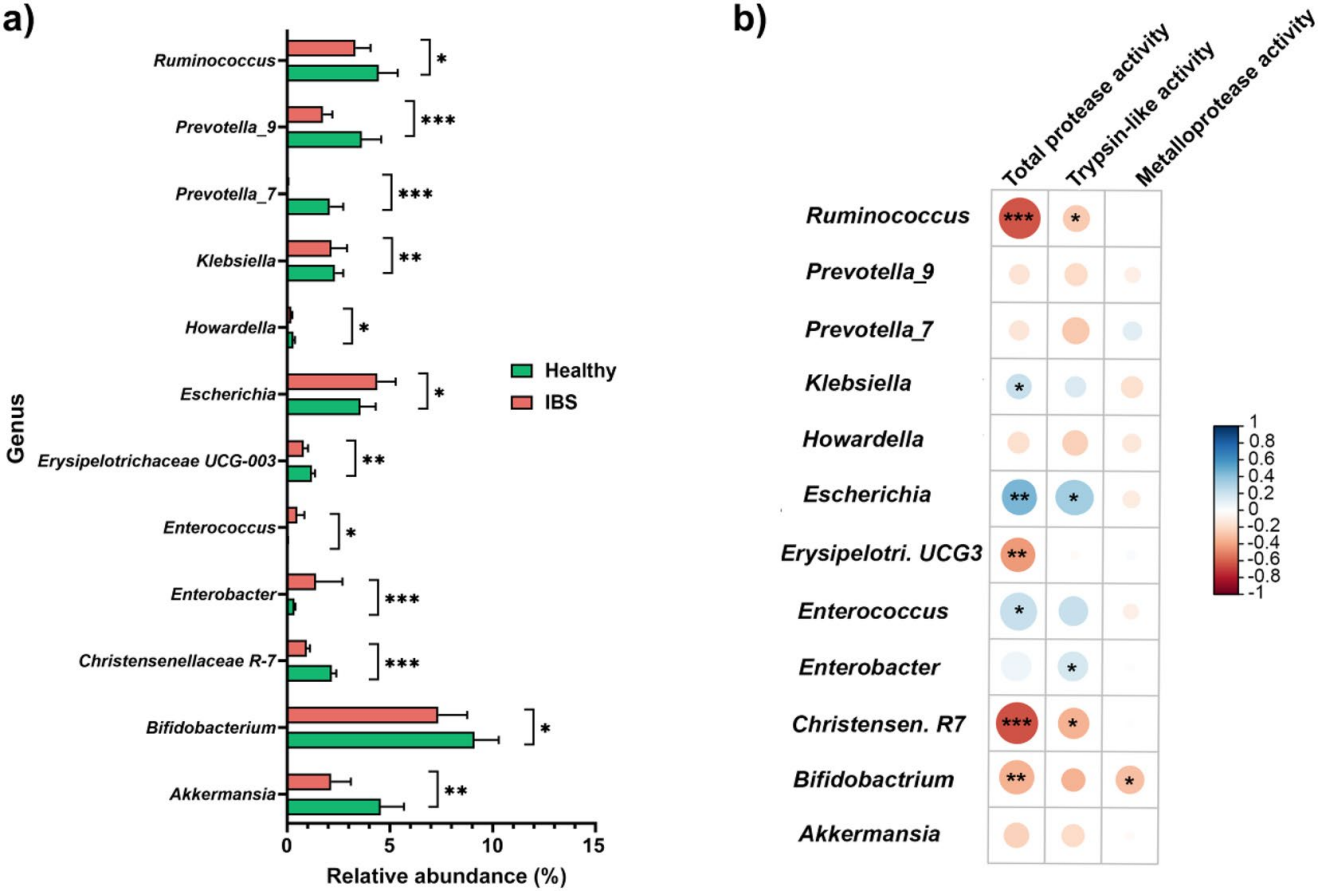
(**a**) Differentially abundant bacterial communities at genera level from IBS samples (red) compared to healthy controls (green). (**b**) Association between abundant bacterial genera and differentially upregulated proteolytic activities. *Escherichia*, *Enterococcus* and *Klebsiella* genera are positively associated to the total protease activity as well as Trypsin-like activity. *Bifidobaterium* was the only genus negatively correlated to metalloproteases activity. Statistical analysis was achieved using the Mann Whitney test. *p < 0.05, **p < 0.01, ***p < 0.001.

At genus level, as shown in Fig. 6a, various genera were under-represented in fecal samples of IBS patients compared to healthy controls. *Bifidobacterium, Ruminococcus, Akkermansia, Erysipelotrichacea UCG-003* and *Christensenellaceae R-7* genera are highly decreased in IBS group. From these depleted genera, several bacterial strains including *Bifidobacterium longum* and *Akkermensia municiphila* has been widely reported for their anti-inflammatory properties through the production of short chain fatty acids such as butyrate. Furthermore, although *Ruminococcus* genera members are also known producers of saturated fatty acids, two strains, namely *Ruminococcus gnavus* and *Ruminococcus torques*, have been associated with IBS symptoms^63^. Besides, *Prevotella_7* and *Prevotella_9*, two genera known to be beneficial and associated with gut wellbeing, were significantly decreased in IBS samples compared to healthy subjects (*p* < 0.001). In this context, a negative correlation between an increase of *Prevotella* and the risk of IBS diarrheal phenotype was previously reported^64^. However, the recent study from Lo Presti et al. did not find such a correlation between *Prevotella* and IBS subtype highlighting the need to use a large multicenter cohort of IBS patients^65^. Interestingly, IBS patients were characterized by an overabundance of genera including *Escherichia, Enterococcus* and *Enterobacter*, considered as pathogenic. Consistent with the findings of this study, previous studies have also found lower fecal *Bifidobacterium* and higher levels of *Escherichia, Enterococcus* and *Enterobacter* in patients with IBS versus healthy controls^22,54,60^. Moreover, the decrease of *Erysipelotry UCG3* reported in our study agrees with the lower abundance of *Erysipelotrichaceae* previously found in IBS-M and IBS-D patients^60^.

### Gut microbiota association with differentially upregulated proteolytic activities

To investigate the relationship between increased proteolytic activities and altered gut microbiota, we performed a correlation analysis. It revealed a positive correlation between total proteolytic activity and 3 microbial genera, namely *Escherichia*, *Enterococcus*, and *Klebsiella* (Fig. 6b). Interestingly, *Escherichia* and *Enterobacter* were also positively correlated to the increased trypsin-like activity in IBS patients. Such pathogenic bacteria encode for a virulence factor named serine protease autotransporters of *Enterobacteriaceae* (SPATE) harboring a trypsin-like serine protease domain*.* Moreover, we observed a significant negative correlation between *Ruminococcus*, *Erysipelotrichacea* UCG-003, *Bifidobacterium*, and *Christensenellaceae* R-7 genus and the total proteolytic activity increase. *Ruminococcus* and *Christensenellaceae R-7* alteration were likely negatively correlated with trypsin-like activity increase. It was previously shown that the *Ruminococcaceae* family has a negative correlation with fecal protease activity in IBS, which agrees with our findings^66^. Concerning *Bifidobacterium,* it was the only genus negatively correlated to metalloprotease activity. Of interest, some *Bifidobacterium* strains have been shown to downregulate the expression of metalloproteases in cancer and skin photoaging^67,68^. It will then be interesting to further investigate the relationship between gut microbiota and metalloprotease activities in the context of IBS notably through a further characterization of upregulated proteases using proteomic approaches.

## Conclusion

This study has reported the profiling of fecal proteolytic activities in the first Tunisian cohort of IBS-M patients and has shown a marked increase in serine protease activity in IBS patients, especially trypsin-like activity. For the first time, we showed an increase in PR3 and metalloproteinase activities in IBS-M patients. Even though the proteolytic activity of substrates assumed to be specific for PR3, elastase, cathepsin G, and metalloproteases are increased in patients with IBS-M, is it not possible to conclusively rule out the possibility of non-specific cleavage of the substrate by some other protease and that confirming the identity of the exact proteases that are up-regulated in IBS-M should await future proteomic analysis. This study also provides new insights on the alteration of the gut microbiota composition and its potential role in proteolytic imbalance in IBS-M. Although in this study we did not investigate the origin of up-regulated serine- and metallo- proteases, IBS symptoms result from both proteolytic-mediated and microbiota-mediated alteration of the epithelial barrier. Evidence suggests the existence of an overlap between host and microbial protease activities and their strong impact on intestinal barrier homeostasis. Larger and multicenter cohorts of IBS patients are required to further investigate the association between proteolytic profiles, microbiome profiles and IBS symptoms in African populations compared to occidental ones. Demographic, genetic and dietary factors should be included to assess their impact on the gut wellbeing in the case of IBS. In addition, further analyses are needed to shed light on the mechanistic mode of action of over-abundant proteases and gut microbiota dysbiosis in IBS disorder. This knowledge cohort will provide fundamental insight on the host-microbiota interactions in the IBS context and can constitute with the advancement of knowledge, a new avenue to develop novel therapeutic strategies aiming the treatment of IBS.

## Data Availability

The datasets used and/or analyzed during the current study are available from the corresponding author on reasonable request.
